# First Detection and Circulation of RHDV2 in New Zealand

**DOI:** 10.3390/v16040519

**Published:** 2024-03-28

**Authors:** Robyn N. Hall, Katherine Trought, Tanja Strive, Janine A. Duckworth, Maria Jenckel

**Affiliations:** 1 CSIRO Health & Biosecurity, Acton, ACT 2601, Australia; 2 Centre for Invasive Species Solutions, Bruce, ACT 2617, Australia; 3 Ausvet Pty Ltd., Fremantle, WA 6160, Australia; robyn.hall@ausvet.com.au; 4 Manaaki Whenua-Landcare Research, Lincoln 7608, New Zealand; troughtk@landcareresearch.co.nz (K.T.); duckworthj@landcareresearch.co.nz (J.A.D.)

**Keywords:** European rabbit, *Oryctolagus cuniculus*, invasive species, RHDV2, viral pathogens

## Abstract

Rabbit haemorrhage disease virus 2 (RHDV2) is a highly pathogenic lagovirus that causes lethal disease in rabbits and hares (lagomorphs). Since its first detection in Europe in 2010, RHDV2 has spread worldwide and has been detected in over 35 countries so far. Here, we provide the first detailed report of the detection and subsequent circulation of RHDV2 in New Zealand. RHDV2 was first detected in New Zealand in 2018, with positive samples retrospectively identified in December 2017. Subsequent time-resolved phylogenetic analysis suggested a single introduction into the North Island between March and November 2016. Genetic analysis identified a GI.3P-GI.2 variant supporting a non-Australian origin for the incursion; however, more accurate identification of the source of the incursion remains challenging due to the wide global distribution of the GI.3P-GI.2 variant. Furthermore, our analysis suggests the spread of the virus between the North and South Islands of New Zealand at least twice, dated to mid-2017 and around 2018. Further phylogenetic analysis also revealed a strong phylogeographic pattern. So far, no recombination events with endemic benign New Zealand rabbit caliciviruses have been identified. This study highlights the need for further research and surveillance to monitor the distribution and diversity of lagoviruses in New Zealand and to detect incursions of novel variants.

## 1. Introduction

Rabbit haemorrhagic disease virus 2 (RHDV2, or genotype GI.2 [1]) is a lagovirus in the family *Caliciviridae* that first emerged in France in 2010 [2,3]. Like the closely related RHDV1 (genotype GI.1c), it causes an acute, fulminant hepatitis and severe multisystemic inflammatory response leading to disseminated intravascular coagulation and death within 36–72 h after infection [4]. However, unlike RHDV1, RHDV2 is lethal in young rabbits that are typically only subclinically infected with RHDV1 [2,5]. Moreover, apart from European rabbits (*Oryctolagus cuniculus*), RHDV2 can infect other lagomorphs, such as hares and jackrabbits (*Lepus* sp.) and cottontails (*Sylvilagus* sp.) [1,6,7,8,9,10,11,12,13,14].

**Figure 1:**
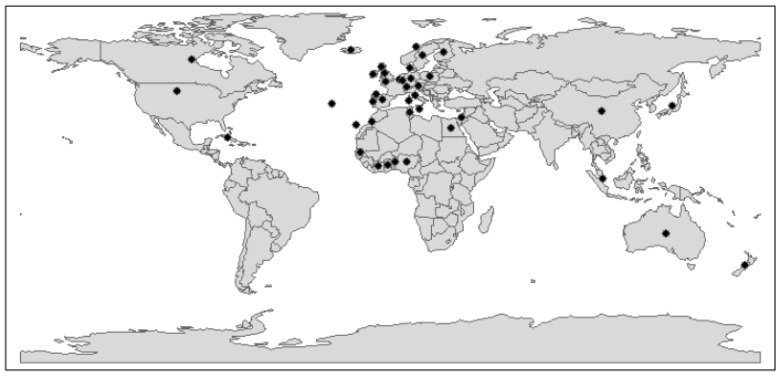
RHDV2 incursions globally, 2010 to 2021. Black dots represent countries reporting RHDV2 incursions since its emergence in 2010. Adapted from [44].

RHDV2 is truly an emerging, panzootic virus. Between 2010 and 2021, RHDV2 incursions were reported by over 35 countries globally (Figure 1). RHDV2 can (at least partially) overcome immunity against prior RHDV1 infection and vaccination [5,15]; it has a broader host range than RHDV1 [1,6,7,8,9,10,11,12,13,14]; and it appears to enter susceptible rabbit populations earlier than RHDV1 variants [16]. Taken together, these factors begin to explain the rapid spread of RHDV2 and the corresponding epidemiological replacement of the previously dominant RHDV1 variants.

Lagoviruses are small non-enveloped viruses of about 25–40 nm in diameter [17,18,19]. The RHDV genome is about 7.5 kb in size and encodes both structural proteins (VP60 and VP10, which form the virus capsid) and non-structural (NS) proteins (from the 5′-end: p16, p23, helicase, p29, viral protein genome-linked (VPg), protease, and RNA-dependent RNA polymerase (RdRp)) in two open reading frames (ORFs) [20]. The first ORF (ORF1) encodes a large polyprotein that is cleaved by the virus-encoded trypsin-like cysteine protease into mature NS proteins and VP60 [21]. The second ORF (ORF2) encodes only the minor capsid protein VP10. Sub-genomic RNA of around 2 kb in size encodes the capsid proteins VP60 and VP10, which are also organised in two ORFs [22,23,24,25]. Both genomic and sub-genomic RNAs are linked to VPg on the 5′-end and are polyadenylated at the 3′-end [22,26,27]. The presence of genomic and sub-genomic RNAs and sequence homology between the 5′-end and the RdRp/VP60 junction enable frequent recombination with a described breakpoint between the RdRp and VP60 coding region [28].

The RHDV2 taxonomic designation comprises several distinct variants that share the genotype GI.2 structural (S) gene coding sequences but differ in the genotype of their NS gene sequences [28,29]. These variants are described by the nomenclature [x]P-[y], where [x]P refers to the genotype of the polymerase (representative of the NS sequence, based on the nomenclature for human noroviruses [1]), and -[y] refers to the S sequence genotype. Reported RHDV2 variants include the GI.1bP-GI.2 [30,31,32], GI.4eP-GI.2 [33], GI.4cP-GI.2 [28], GI.3P-GI.2 [6,29,34,35,36,37,38,39,40,41,42,43], and GII.1P-GI.2 [39] variants. While all reported RHDV2 variants can lethally infect hares and young rabbits (unlike RHDV1), they appear to differ in epidemiological fitness [28]. However, the precise biological mechanisms underpinning this are yet to be elucidated.

European rabbits are an introduced species in both New Zealand and Australia that cause enormous damage to native ecosystems and agricultural industries [45]. In New Zealand, rabbits compete with livestock for pasture and provide a stable food source for other invasive mammals and carriers of bovine tuberculosis, such as ferrets, stoats, and feral cats [46,47]. Rabbits graze on many vulnerable native plant species and damage erosion-prone soils through burrowing and digging activity. In Australia, rabbit caliciviruses have been used to help manage feral rabbit populations since 1995. Subsequently, in 1997, RHDV1 was illegally imported and released in New Zealand [48]. Following the official approval of RHDV1 in New Zealand, this variant was observed to reduce rabbit numbers at release sites, making it an effective measure for rabbit population control there [49].

Prior to the detection of RHDV2 in 2018, three lagovirus variants were known to be present in New Zealand. Two variants serve as biological controls to manage the population of pest rabbits, RHDV1 (V351 Czech, genotype GI.1cP-GI.1c), introduced in 1997 [48], and RHDV1a (K5, genotype GI.1aP-GI.1a), present since 2018 [50]. Additionally, a benign enterotropic rabbit calicivirus (genotype GI.4P-GI.4) has circulated subclinically in wild rabbit populations since the early 1990s [51]. This non-pathogenic variant can induce partial immunological cross-protection to lethal RHD, reducing the effectiveness of rabbit biocontrol programs in regions with high seroprevalence. Then, in April 2018, RHDV2 was detected in New Zealand for the first time [50]. In this work, we describe the field and molecular epidemiological characteristics of this incursion.

## 2. Materials and Methods

### 2.1. Tissue Sample Collection

Wild and domestic rabbit liver samples (*n* = 195) were collected from various locations across the North and South Islands of New Zealand between 2017 and 2021. Samples were collected opportunistically from the carcasses of rabbits found dead by council staff and members of the public. No animal ethics approvals are required for sampling rabbits that are found dead in New Zealand [50]. Approximately 100–200 mg of liver tissue was subsampled into 1 mL of RNAlater buffer (ThermoFisher Scientific, Waltham, MA, USA), transported at ambient temperature to Manaaki Whenua-Landcare Research molecular laboratory in Lincoln, and then stored at −80 °C until analysis.

### 2.2. Virus Detection

Total RNA was extracted from 20–50 mg of liver tissue using the iNtRON easy-spin™ [DNA free] Total RNA Extraction Kit (iNtRON Biotechnology, Seongnam, Kyonggi-do, Republic of Korea). cDNA was synthesised from the extracted RNA using SuperScript IV Reverse Transcriptase and Oligo d(T)_20_ (Life Technologies Corporation, Carlsbad, CA, USA). Samples were screened for the presence of lagovirus RNA with a multiplex PCR using KAPA2G Robust HotStart PCR Kit (Kapa Biosystems, Inc., Wilmington, MA, USA), as previously described [33] (Table 1). PCR products were visualized on a 2% agarose gel stained with SYBRsafe (ThermoFisher Scientific).

### 2.3. Virus Sequencing

Total RNA (20 μL) from lagovirus-positive liver samples (*n* = 52) was precipitated with a 0.1 volume of 3M sodium acetate and 3 volumes of 100% ethanol for transportation, and RNA pellets were later resuspended in nuclease-free water prior to PCR amplification. RNAs were reverse-transcribed using Superscript IV Reverse Transcriptase (Thermofisher Scientific) and an Oligo d(T)_18_ primer (Sigma-Aldrich, St. Louis, MI, USA), according to the manufacturer’s directions. Overlapping primer pairs (Table 2) specific for GI.3P-GI.2 were used to amplify the viral genome in five fragments, and libraries were prepared and sequenced using Illumina MiSeq technology (MiSeq reagent kit v2 300-cycles) (Illumina, San Diego, CA, USA), as described previously [52,53].

### 2.4. Phylogenetic and Phylogeographic Analyses

The quality of raw sequencing data was assessed using FastQC (v. 0.11.8). Adapter and primer sequences, as well as low-quality regions, were trimmed using Trimmomatic (v.0.38) [55], and overlapping reads were merged using FLASh (v1.2.11) [56]. Cleaned sequence reads from each isolate were mapped to the rabbit hemorrhagic disease virus-FRG complete genome (GenBank accession M67473.1) using the Geneious mapper as implemented in Geneious Prime 2021.1.1, and consensus sequences were extracted.

Representative global lagovirus sequences were selected by retrieving all near-complete lagovirus sequences from the NCBI Nucleotide (nt) database (as of 8 September 2021) and identifying clusters with a 95% sequence identity cut-off using CD-HIT-EST [57]. Sequences without sufficient temporal data were removed from the dataset. Newly sequenced New Zealand RHDV2 genomes were aligned with these representative global sequences using the FFT-NS-2 algorithm within MAFFT v7.450 [58]. Maximum likelihood (ML) phylogenies (*n* = 179) were estimated separately for the NS and S gene regions (nucleotide positions 28–5304 and 5305–7382, respectively, based on GenBank accession M67473.1) using IQTREE v1.7.0b7 [59], with the best-fit model as determined by ModelFinder [60]. Branch support was estimated using 1000 ultrafast bootstrap replicates [61] and 1000 replicates of the SH-aLRT test [62]. Phylogenies were rooted at the midpoint between the genogroup I and II clades. We were not able to amplify the 5′-end of three of the New Zealand RHDV2 sequences (OM372659, OM372625, and OM372627); NS sequence phylogenies were estimated both with and without these sequences, and we observed no significant changes in topology; therefore, analyses were conducted with these sequences included.

To reveal any geographic clustering and structure, a maximum likelihood phylogeny was also inferred separately for the 52 New Zealand RHDV2 NS sequences, and the tips were matched to their location on a map of New Zealand.

### 2.5. Bayesian Evolutionary Analysis

All New Zealand RHDV2 sequences and global lagovirus sequences were aligned, and phylogenetic trees were inferred as described above. Additionally, a phylogenetic tree was constructed as described above for a near-full genome alignment. A linear regression of root-to-tip distances against sampling times, as implemented in TempEst [63], was used to determine sufficient temporal signal. The alignment of non-structural genes was found to have the strongest temporal signal (correlation coefficient = 0.53) and was thereafter used for all subsequent analyses. A time-scaled phylogeny was inferred by using a Bayesian Markov chain Monte Carlo (MCMC) approach. To assess the most appropriate clock prior (strict versus uncorrelated log-normally distributed (UCLD)) and tree prior (Gaussian Markov random field Bayesian Skyride model versus constant size coalescent versus exponential coalescent), we used the marginal likelihood estimations (MLEs) (path sampling/stepping-stone sampling) as implemented in BEAUti (v1.10.4) [64]. Substitution model GTR + F + I + G4 was used for all MLEs. The best MLE values were reached with a strict clock and exponential coalescent with a chain length of 20 million and were subsequently used for a Bayesian Evolutionary Analysis Sampling Tree (BEAST) analysis (v1.10.4). The analysis was run twice to convergence (ESS > 200) to confirm consistency. TreeAnnotator (v1.10.4), available in the BEAST package, was used to create a maximum clade credibility (mcc) tree from the Bayesian phylogenetic inference results with a 10% burn-in.

### 2.6. Figures

All figures were generated in R v4.2.1 [65] using the following packages: ggtree v3.3.0.900 [66], scales v1.1.1 [67], tidyverse v1.3.1 [68], spData v2.0.1 [69], ggspatial v1.1.5 [70], sf v1.0-5 [71], cowplot v1.1.1 [72], ggplot2 (v3.4.2) [73], dplyr (v1.1.1) [74], RColorBrewer (v.1.1-3) [75], ggthemes (v4.2.4) [76], and treeio (v.1.24.1) [77].

## 3. Results

### 3.1. RHDV2 Was First Detected in New Zealand in 2017

**Figure 2:**
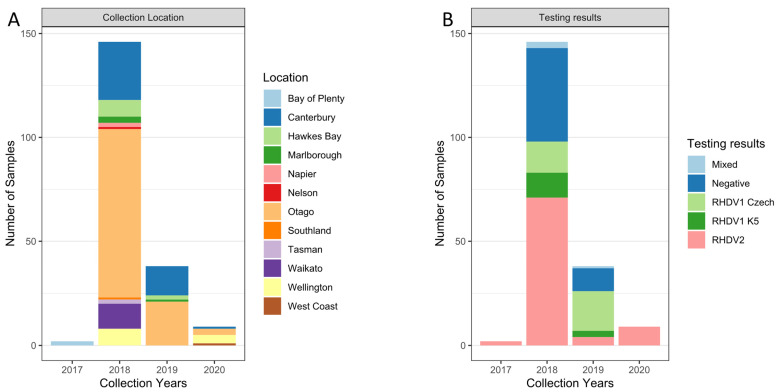
Stacked bar charts of tested samples from 2017 to 2020. Samples are coloured by (**A**) the location of collected samples and (**B**) testing results by RT-PCR per year.

Routine sampling of dead rabbits was previously conducted in New Zealand to monitor the impacts of the existing biocontrols, RHDV1 (since 1997), and the more recently introduced RHDV1a-K5 (since March 2018). Between 2017 and 2020, 195 rabbit liver samples were tested for the presence of lagoviruses, the majority of those samples in 2018 (*n* = 146), using a multiplex RT-PCR. Samples were collected from across New Zealand, with most of them originating from the South Island’s Otago region (*n* = 105) (Figure 2).

**Figure 3:**
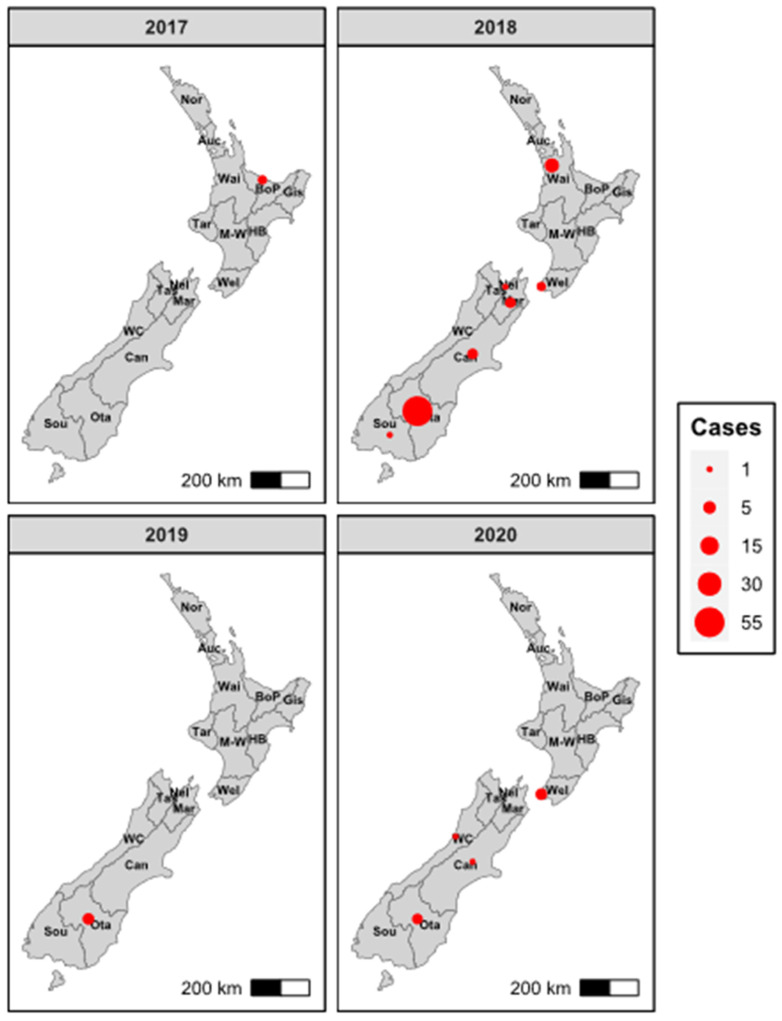
Detections of RHDV2 in New Zealand from 2017 to 2020. RHDV2 was detected using RT-PCR in samples collected from wild and domestic rabbits found dead. Plots are faceted by collection year. Dot sizes correlate with case numbers. Nor—Northland, Auc—Auckland, Wai—Waikato, BoP—Bay of Plenty, Gis—Gisborne, Tar—Taranaki, M-W—Manawatu-Wanganui, HB—Hawkes Bay, Wel—Wellington, Nel—Nelson, Tas—Tasman, Mar—Marlborough, WC—West Coast, Can—Canterbury, Ota—Otago, Sou—Southland.

RHDV2 was first detected in New Zealand in a liver sample from a wild adult European rabbit found dead on 6 April 2018 in the Awatere Valley, Marlborough, in the northern point of the South Island (Figure 2) [78]. However, following this detection, retrospective testing of previously collected samples identified RHDV2 in two wild rabbit samples collected from a single farm from the Bay of Plenty region in the North Island in December 2017 (Figure 2 and Figure 3), preceding the original April 2018 sample collection date. RHDV2 was subsequently detected in 71 samples in 2018 (March, April, June, and August to November), in 4 samples in 2019 (April, June, and December), and in 9 samples in 2020 (January, April, October, and December) across New Zealand’s North and South Islands (Figure 2 and Figure 3). Mixed testing results suggesting co-infection with multiple lagoviruses were observed in three samples in 2018 and one sample in 2019 (Figure 2B), which were positive for both the RHDV2 and RHDV1-V351 Czech variants.

### 3.2. Phylogenetic Analysis Reveals the New Zealand RHDV2 Incursion as Genotype GI.3P-GI.2

On average, a total of 305,675 cleaned sequencing reads were generated per sample, with around 75.3% of those mapping to the respective isolates. This resulted in genome coverage between 2872× and 5726× (i.e., very deep coverage) (Supplementary Table S1). Subsequent data analysis led to the generation of 49 near-complete NZ RHDV2 consensus genomes (GenBank accession: OM372617–24, OM372626, OM372628–58, and OM372660–OM372668) and three partial-consensus genomes (GenBank accession: OM372659, OM372625, and OM372627). The nucleotide identity between the consensus genomes ranged from 97.98 to 100%.

**Figure 4:**
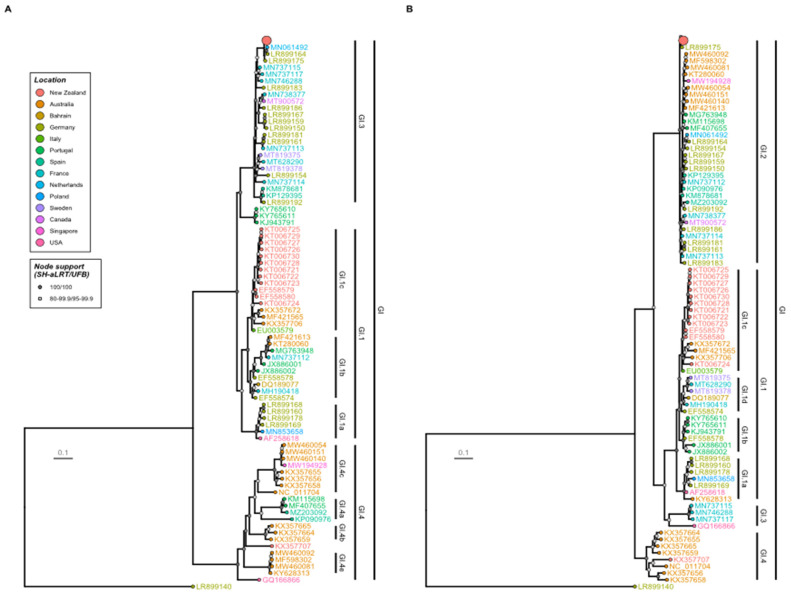
Maximum likelihood phylogenies of New Zealand and representative global RHDV2 sequences. All near-complete lagovirus genomes were downloaded from GenBank, and representative sequences spanning the known diversity were derived using CD-HIT-EST. These were aligned with the New Zealand RHDV2 sequences. Phylogenies were inferred for the (**A**) non-structural and (**B**) structural genes independently. Taxa are coloured by country of origin, and the genogroup, genotype, and variant type are indicated by clade labels. Strong (100) and moderate (80–99) clade support is indicated by the filled dark grey and light grey circles, respectively, at internal nodes. The scale bar represents substitutions per site. The clade for New Zealand samples (this study) has been collapsed and is shown as a big, red-filled dot. The collapsed New Zealand clade for the non-structural (**A**) genes is shown in Figure 5.

Phylogenetic analyses revealed that the New Zealand RHDV2 sequences formed monophyletic clades in both the NS and S gene phylogenies, suggesting a single incursion event into the country with subsequent ongoing spread (Figure 4). Specifically, the variant was identified as a recombinant GI.3P-GI.2 lagovirus. BLASTn analyses showed that all 52 New Zealand RHDV2 sequences were most similar to LR899175, a GI.3P-GI.2 virus recovered from a domestic rabbit from the Rhineland-Palatinate region of Germany in 2014 [39]. The New Zealand sequences shared 96.8–97.9% nucleotide identity with this virus across the whole genome. Importantly, the New Zealand GI.3P-GI.2 incursion is clearly unrelated to both the GI.1bP-GI.2 incursion into Australia [30] and the subsequent GI.4cP-GI.2 and GI.4eP-GI.2 recombination events within Australia [28,33].

**Figure 5:**
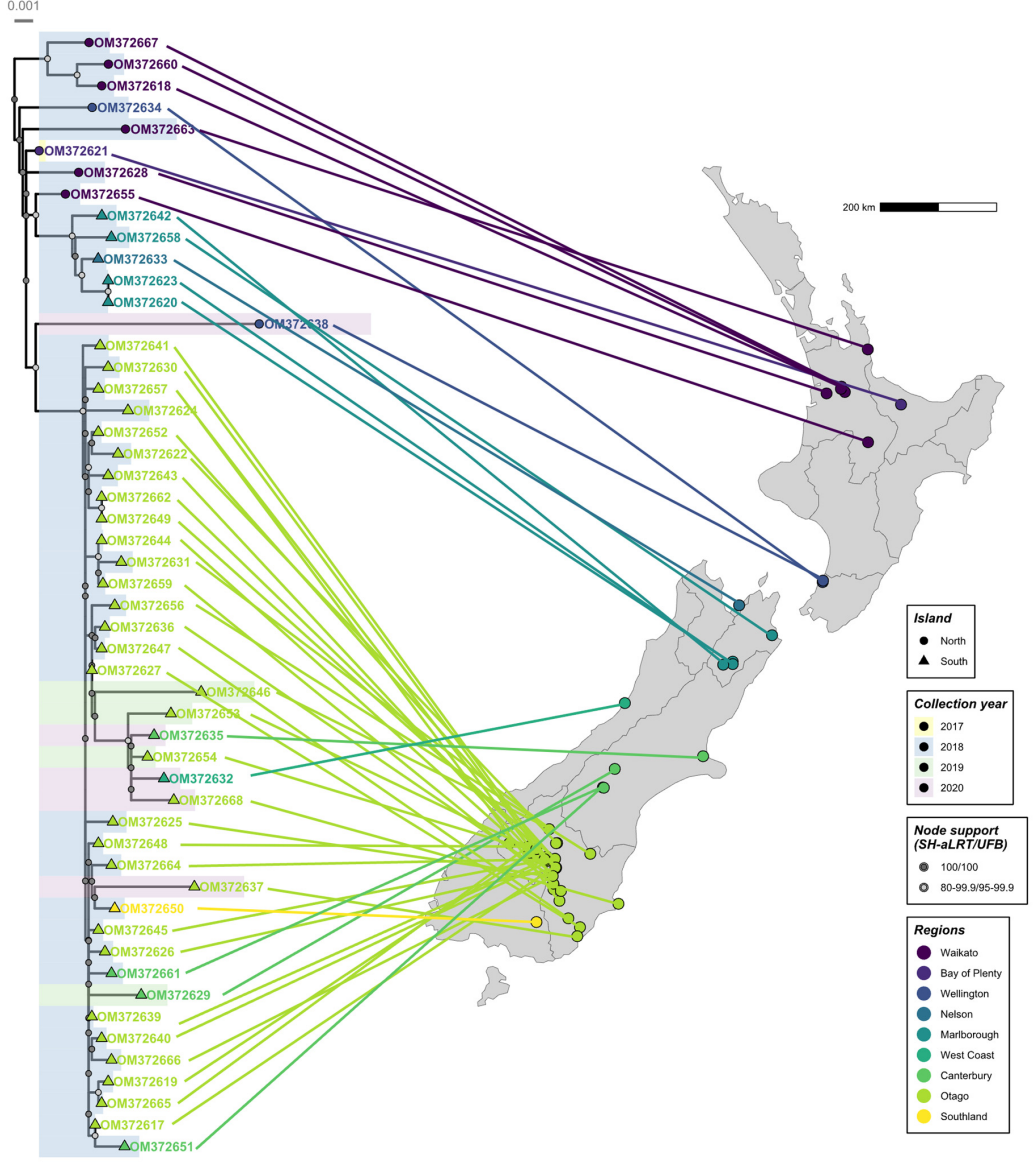
Maximum likelihood phylogeny and phylogeographic analysis of New Zealand RHDV2 non-structural genome sequences. A phylogeny was inferred for the 52 non-structural New Zealand RHDV2 sequences. Taxa names are coloured by region of sample collection, and branches are highlighted by collection year. Tip points represent whether samples were collected in the North or the South Island. Strong and moderate clade support is indicated by the filled dark grey and light grey circles, respectively, at internal nodes. The scale bar represents substitutions per site. Lines connect the sample location to the location on the New Zealand map.

Amongst the New Zealand RHDV2 sequences, there was a clear pattern of increasing genetic diversity over time (Figure 5), further supporting a single introduction with ongoing transmission. Several 2020 sequences have comparatively long branch lengths, most likely suggesting substantial undetected spread in New Zealand rabbit populations. A clear phylogeographic signal was also observed despite the heavy sampling bias towards the South Island. Most North Island sequences clustered close to the root of the New Zealand phylogeny, correlating with the earliest detections in the North Island. Sequences from Nelson and Marlborough (adjacent regions in the north of the South Island) formed a monophyletic clade, as did sequences from the southern regions of the South Island, suggesting at least two instances of spread across the Cook Strait.

### 3.3. First Introduction to New Zealand in Mid-2016

**Figure 6:**
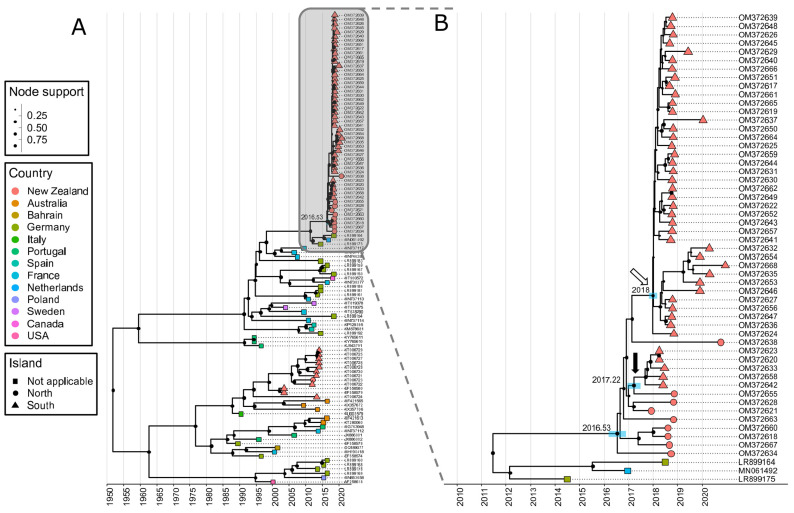
Time-resolved phylogenetic analysis of New Zealand and global representative RHDV2 genome sequences. A time-resolved phylogeny was inferred for the non-structural protein genes of global RHDV2 sequences (**A**) and the New Zealand (NZ) clade (**B**). The NZ clade is shaded in grey in (**A**). Tips are coloured by the country of origin, and node support is indicated by the size of dots at the internal nodes. Blue horizontal bars refer to the 95% highest posterior density (HPD) with the median displayed at the respective nodes; shown are the time of introduction into NZ and inferred spread events (first event indicated by black-filled arrow and second event indicated by white-filled arrow) from the North and South Island. The *x*-axis is given in years.

Time-resolved phylogenetic analysis of 52 RHDV2 samples from New Zealand and representative global sequences suggests the introduction of RHDV2 into New Zealand around mid-2016 (95% HPD 2016.17–2016.86) (Figure 6), implying that the virus spread undetected after arrival for approximately 1 year. The first spread event from the North to the South Island could be dated to early to mid-2017 (95% HPD 2016.96–2017.49), shortly after the virus arrived in New Zealand (black arrow, Figure 6). A second spread event likely occurred around 2018 (95% HPD 2017.82–2018.17) (white arrow, Figure 6).

## 4. Discussion

The global spread of RHDV2 since its emergence in 2010 has been dramatic, resulting in epizootic lagomorph mortality events on at least five continents and in several island populations. With the expanded host range of RHDV2, it is now considered to be endemic in wide parts of the world in wild lagomorph populations. The stability of infectious viruses in the environment [79], along with the extremely high incidence and high viral loads observed in infected animals (leading to shedding large amounts of virus into the environment), indicate that fomite transmission, mechanical transmission via insect vectors [80], and/or the movement of infected carcasses via scavengers [31,40,81,82,83] are likely key contributors to long-distance spread.

While RHDV1 has been endemic in New Zealand since 1997 [48], New Zealand remained free of RHDV2 until its first detection in 2018, with subsequent retrospective detection to 2017 as described in this study. Here, we show that a single incursion event, probably into the North Island in 2016 or 2017, led to the establishment of RHDV2 in New Zealand’s wild and domestic rabbit populations. While the earliest detection was from December 2017, there was limited sampling of wild rabbits in New Zealand for molecular testing prior to 2017. Phylodynamic analysis supports a recent incursion, with a most likely incursion date between March and November 2016. It is likely that RHDV2 underwent cryptic spread for approximately one year before the initial detection. This is not overly surprising since the sampling of wild rabbit carcasses is opportunistic and biased. Carcasses can be difficult to recover when infected rabbits die in inaccessible locations, carcasses may be taken by scavengers, and someone must be willing to collect and submit a sample for analysis.

Phylogenetic analyses showed that the New Zealand RHDV2 variant was a GI.3P-GI.2 virus that was previously detected in Spain [6,34,35,36], the Netherlands [38], Germany [39], France [29], Portugal [34,37], Poland [40], the USA [43], Canada [42], and China [41]. The widespread distribution of this variant makes the attribution of the source of the New Zealand incursion highly uncertain. However, despite extensive genetic analyses, the New Zealand RHDV2 variant (GI.3P-GI.2) virus has not been detected in Australia [28,84], where phylodynamic and serological studies have demonstrated RHDV2 circulation since 2014 [85,86], suggesting that, to date, there has been no exchange of RHDV2 variants between the two countries despite their relative geographical proximity. The GI.3P-GI.2 variant was not detected in a recent Australian study of lagoviruses sampled up to December 2022 [84].

Since the incursion of RHDV2 into New Zealand, phylogenetic analysis suggests ongoing spread throughout New Zealand rabbit populations and at least two spread events from the North to the South Island. The North and South Islands are separated by the Cook Strait, a distance of 23 km at its narrowest point. Transmission across the Strait may have been human-mediated, via fomites, through the self-directed or passive/wind-assisted movement of mechanical vectors such as flies, or perhaps via raptors or scavenging birds. Other authors have suggested similar routes for the incursion of lagoviruses onto and/or between islands [31]. The limited scope of the available epidemiological data precludes a more comprehensive analysis of the spread mechanisms of RHDV2 in New Zealand. Importantly, in response to the incursion of RHDV2 in New Zealand, Agricultural Compound and Veterinary Medicines approved the importation of the bivalent Filavac VHD K C+K vaccine for the immunisation of domestic rabbits against RHDV1 and RHDV2. This enables pet owners, breeders, and meat producers to protect domestic rabbits from both RHDV1 and RHDV2 virus variants. Previously, only the monovalent Cylap RHDV1 vaccine was available in New Zealand.

Elsewhere in the world, RHDV2 has rapidly replaced previously dominant RHDV1 variants [39,84,86]. Likewise, in New Zealand, RHDV2 was the only variant detected in 2020, with the pathogenic RHDV1 Czech and K5 variants only detected until the end of 2019 (Figure 2B). However, it must be noted that the number of samples analysed in 2020 was relatively small compared with previous years. A large number of samples were submitted in 2018 in association with the coordinated national release of the biocontrol variant RHDV1-K5 and a public awareness campaign. However, sample submission declined following the release.

In contrast with Australian observations, no recombination events between RHDV2 and circulating benign rabbit caliciviruses have been identified in New Zealand so far. While multiple recombination events were detected in Australia in the 2-year timeframe following the first detection of RHDV2 [28], all sequenced New Zealand RHDV2 viruses in this study belonged to the GI.3P-GI.2 variant. A small number of samples tested positive for both RHDV2 and RHDV1-V351, indicating co-infection, which is the first step necessary for recombination. Ongoing testing and sequencing are required in the future to monitor changes in viral co-circulation patterns and to detect possible recombination events between lagoviruses in New Zealand. This study highlights the importance of surveillance and monitoring of circulating lagoviruses in New Zealand for the detection of novel variants and to make informed decisions about the deployment of biocontrol agents.

## Figures and Tables

**Table 1:** Primers used for virus detection (from [33]).

Primer	Sequence (5′-3′)	Amplicon Size (bp)	Nucleotide Position ^a^	Gene ^b^
GI.1a-Aus_fwd	GCGTGGCATTGTGCGCAGCATC	562	4349	RdRp
GI.1a-Aus_rev	TGTTGGTGATAAGCCATAATCGCG		4911	
GI.1c_fwd	AGCAAGACTGTTGACTCAATTTCG	435	5995	VP60
GI.1c_rev	AGGCCTGCACAGTCGTAACGTT		6430	
GI.2_fwd	TTTCCCTGGAAGCAGTTCGTCA	336	6213	VP60
GI.2_rev	TGTTGTCTGGTTTATGCCATTTGC		6549	
GI.1a-K5_fwd	TTTATAGATGTATGCCCGCTCAAC	263	4930	RdRp
GI.1a-K5_rev	CCGTTCGAGTTCCTTGCGGACG		5193	

^a^ Nucleotide positions are based on GenBank accession KT280060. ^b^ RdRp: RNA-dependent RNA polymerase coding region; VP60: VP60 capsid protein-coding region (Figure 1).

**Table 2:** Primers used in this study.

Primer	Sequence (5′-3′)	Fragment	Ref
RHDV-1	GTGAAARTTATGSCGGCTATGTCGCGC	1	[52]
GI.3PGI.2_1667R	TGGTCAAGGCCAAAGTTRATCG		This study
RHDV1_GI.3	GTGAAAGTTATGGCGGCTATGT	1 alternate	This study
GI.3PGI.2_1698R	AGCCACTTCCTCTCCTGTGTAT		This study
GI.3PGI.2_1451F	ACAACCAACACCTYGGAATCTTGAATG	2	This study
GI.3PGI.2_3064R	CTTGCCACTCRTCGTACTCRTC		This study
GI.3PGI.2_2918F	TGTGYGCAAACCACCTTGTYAA	3	This study
GI.3PGI.2_4607R	CTCTTGGTGAGTTCCGTCTGYTCA		This study
GI.3PGI.2_4399F	AAGATGGTCGCACGGTTTG	4	This study
GI.3PGI.2_6030R	GAGATCTGCGGGCGAGAT		This study
GI.3PGI.2_5867F	CCACGTTGGTCCTGAGCGTYTA	5	This study
RHDV-end	TTTTTTTTTTTTTTTTTTTTTTTTTTTTTATAATTTACTCTAAATTATAAACCAATTAAATTAATTAAC		[54]

## Data Availability

All sequences generated in this study were deposited in GenBank (Accession numbers: OM372617-OM372668).

## Supplementary Materials

The following supporting information can be downloaded at https://www.mdpi.com/article/10.3390/v16040519/s1: Supplementary Table S1 contains a summary of sequencing data pre- and post-quality control and an overview of mapping results to the rabbit haemorrhagic disease virus-FRG genome (GenBank accession M67473.1).
